# Transmissibility and temporal changes of 2009 pH1N1 pandemic during summer and fall/winter waves

**DOI:** 10.1186/1471-2334-11-332

**Published:** 2011-12-02

**Authors:** Ying-Hen Hsieh, Kuang-Fu Cheng, Trong-Neng Wu, Tsai-Chung Li, Chiu-Ying Chen, Jin-Hua Chen, Mei-Hui Lin

**Affiliations:** 1 Department of Public Health, China Medical University, Taichung 40402, Taiwan; 2 Biostatistics Center, China Medical University, Taichung 40402, Taiwan; 3 Graduate Institute of Biostatistics, China Medical University, Taichung 40402, Taiwan; 4 Center for Infectious Disease Education and Research, China Medical University, Taichung 40402, Taiwan

## Abstract

**Background:**

In order to compare the transmissibility of the 2009 pH1N1 pandemic during successive waves of infections in summer and fall/winter in the Northern Hemisphere, and to assess the temporal changes during the course of the outbreak in relation to the intervention measures implemented, we analyze the epidemiological patterns of the epidemic in Taiwan during July 2009-March 2010.

**Methods:**

We utilize the multi-phase Richards model to fit the weekly cumulative pH1N1 epidemiological data (numbers of confirmed cases and hospitalizations) as well as the daily number of classes suspended under a unique "325" partial school closing policy in Taiwan, in order to pinpoint the turning points of the summer and fall/winter waves, and to estimate the reproduction numbers R for each wave.

**Results:**

Our analysis indicates that the summer wave had slowed down by early September when schools reopened for fall. However, a second fall/winter wave began in late September, approximately 4 weeks after the school reopened, peaking at about 2-3 weeks after the start of the mass immunization campaign in November. R is estimated to be in the range of 1.04-1.27 for the first wave, and between 1.01-1.05 for the second wave.

**Conclusions:**

Transmissibility of the summer wave in Taiwan during July-early September, as measured by R, was lower than that of the earlier spring outbreak in North America and Europe, as well as that of the winter outbreak in Southern Hemisphere. Furthermore, transmissibility during fall/winter in Taiwan was noticeably lower than that of the summer, which is attributable to population-level immunity acquired from the earlier summer wave and also to the intervention measures that were implemented prior to and during the fall/winter wave.

## Background

Although the first known imported case of 2009 pandemic influenza (pH1N1) arrived in Taiwan on May 18 from the U.S. via Hong Kong, Serological evidence has indicated that the pH1N1 virus had spread to central Taiwan by April-June [1]. Local infections and laboratory-confirmed pH1N1 cases in Taiwan started to mount in significant numbers in July-August when the schools were in summer recess. By the time the schools reopened in September, multiple intervention measures had been implemented by the government, which include strict border temperature screening starting in May, a "325" class suspension policy [2,3] implemented in September, and later a mass immunization program [3-5] starting in November. The number of cases began to decline by the end of the year, and continued to do so into early next year, until the government announced on February 23 the end of the fall/winter outbreak [6] with over 3000 laboratory-confirmed cases reported, 910 hospitalizations, and 41 deaths [7].

**Figure 1:**
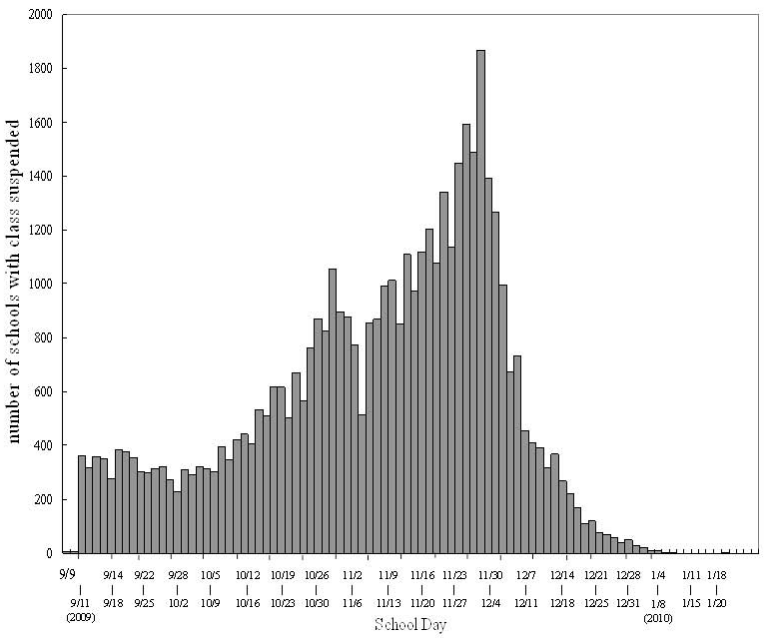
**Daily number of schools (K-12, universities, cram schools, and after school institutes) with at least one class suspended during 9/9/2009-1/1/20/2010 in Taiwan**. (Source: Taiwan CDC Novel Influenza A/H1N1 website).

Although school closing was a widely used method of intervention around the world during the pH1N1 outbreak (see, e.g., [8-13]), its suitability, timing, and the manner of implementation remains controversial. When K-12 schools (kindergarten through high schools) reopened on August 31 in Taiwan, the government implemented a unique partial school closing policy called the "325" class suspension policy aimed toward kindergarten through secondary schools (K-9), cram schools, and after-school institutions. Under this policy, if within any three (3) consecutive school days, two (2) or more students in the same class are diagnosed with influenza, then that class will be suspended for the next five (5) days including weekends and holidays [2,3]. The policy was designed to minimize the potential social impact of full-scale school closings in the event of a major influenza outbreak in the community; to detect cluster infections in school settings early and swiftly; and to contain the infections locally without disruption for the other students in the school. At the height of the class suspensions in late November, more than 1800 classes with more than 50,000 students from almost 800 schools in Taiwan were suspended on a single school day (Figure 1), yet without any visible disruption in the normal functioning of the society.

Moreover, starting November 1, a mass immunization program was initiated in Taiwan sequentially, according to a priority list of 12 target groups [4], with healthcare and public health personnel having the highest priority [5]. Subsequently, preschool children were immunized starting on November 9; and followed by pregnant women, K-6 schoolchildren, and people with major illness/injury being vaccinated starting on November 16; 7-12 year-olds on November 23; and the general population on December 12. By March 16, a total of 5.66 million doses of AdimFlu-S (unadjuvanted H1N1v from Adimmune) or Focetria^® ^(MF59^® ^adjuvanted H1N1v from Novartis) were administered, and more than 5 million of the 23 million Taiwanese had been immunized [14]. Children 12 and under were advised to receive two doses of vaccine, although many of them eventually received only one dose due to various reasons.

A simple mathematical model, the Richards model, is utilized to fit publicly accessible cumulative epidemic data in order to obtain estimates for the turning points (the peaks and volleys of the incidence curve) and the reproduction number R of a particular wave of infections. Examples of applications of the Richards model to infectious diseases include those of SARS [15,16], dengue [17,18], and the 2009 pH1N1 epidemic [19,20]. In this study, we will make use of the Richards model to pinpoint the turning points of each wave of the epidemic, in order to ascertain the temporal changes of the epidemic in Taiwan in the summer months and during the fall and winter days. The transmissibility of the pH1N1 virus during the outbreak is determined through its reproduction number.

## Methods

### Data

The data was accessed from the Central Epidemic Command Center website of the Taiwan Centers for Disease Control (TCDC). Samples were collected from hospitals and clinics participating in the Taiwan Influenza surveillance system under the Taiwan National Influenza Center (Taiwan NIC), which was established in 2006 to integrate all existing efforts of influenza surveillance and notification with laboratory analysis systems throughout Taiwan in order to enhance the epidemic data collection capacity in Taiwan [21]. The weekly laboratory confirmed pH1N1 case data (by the week when the samples were collected and sent to the TCDC-contracted laboratories) and the weekly hospitalization data (by the week the lab-confirmed cases were hospitalized) from June 28, 2009 (epidemiological week or e-week 27 of 2009) to March 27, 2010 (e-week 12 of 2010) was accessed from the weekly Influenza Express made publicly available on the internet by the TCDC [22] during the epidemic. The surveillance protocols in Taiwan remained essentially the same throughout the data period since, by the time the data were collected, clinical characteristics of the pH1N1 infection had already been well understood from the spring outbreaks around the world.

We also accessed the daily record of numbers of classes suspended and number of schools with at least one class suspended during the fall school semester (September 9, 2009 to January 20, 2010) from the TCDC daily pH1N1 updates [23] during the epidemic. The time series of class suspension data is given in Figure 1. Since this data is for school days only, the days are specified in the horizontal axis of Figure 1 in weekly increments of 5 school days, except for weeks with less than 5 school days at the beginning and the end of the school semester as well as the week containing the New Year holiday (January 1).

### The Richards Model

The Richards model [24] is of the form: $C^{'}\left(t\right)=rC\left(t\right)\left[1-{\left(\frac{C}{K}\right)}^{a}\right]$, where the prime symbol "'" denotes the rate of change over time which is in e-weeks. *C(t) *is the cumulative number of cases at time *t *(in weeks), *K *is the cumulative case number over a single wave or phase of outbreak, *r *is the per capita growth rate of the infected population, and *a *is the exponent of deviation. The explicit solution of the equation is $C\left(t\right)=K{\left[1+e^{-ra\left(t-t_{m}\right)}\right]}^{-1∕a}$. Here the parameter *t*_m _is related to the turning point t_i _of a wave (or the inflection point of the cumulative case curve) by the simple formula *t*_*m*_ = *t*_*i*_ + *lna*/*(ra)*, where *ln *denotes the natural logarithm function.

Moreover, *R*_0_ = *exp(rT) *where T is the generation interval of the disease, or the average time interval from the onset of one infected person to the time when the onset of his or her contacts occurs. It has been shown mathematically [25] that, given the growth rate *r*, the expression *R*_0_ = *exp(rT) *provides an upper bound of the basic reproduction number regardless of the distribution of the generation interval that is being used. In this work, we will use the term *effective *reproduction number R instead, due to the community-level immunity likely achieved by July and the interventions implemented during the two waves.

The Richards model is a phenomenological model which can be used to describe the phenomenon of a biological growth (of cumulative number in this case) without requiring detailed information on the actual process of disease transmission. The basic premise of the Richards model is that the incidence curve of a single wave of infections contains a single peak of high incidence, resulting in an S-shaped cumulative epidemic curve and a single turning point (or peak incidence) of the outbreak. The turning point, defined as the point in time at which the rate of accumulation changes from increasing to decreasing, or vice versa in the event of a multi-wave outbreak, can be easily pinpointed by locating the inflection point of the cumulative case curve, i.e., the moment at which the trajectory begins to decline, as demonstrated in previous applications (see, e.g., [15-20]. This quantity has important epidemiologic implications, indicating either the valley (i.e., moment of acceleration after deceleration) or peak (i.e., moment of deceleration after acceleration) of a disease incidence curve. Multi-wave outbreaks also can be modeled by using the multi-phase Richards model [16,18]. Simultaneous estimates of the model parameters r, a, t_i_, and K, based on fitting the explicit solution of the Richards model for *C(t) *to the epidemic data used in the study, can be obtained easily and efficiently using any standard software with a nonlinear least-squares approximation tool, such as SAS or Matlab. The procedure for locating multiple turning points for multi-wave outbreak, which required the use of the multistage Richards model, is detailed in [16] and hence is omitted here.

## Results

**Figure 2:**
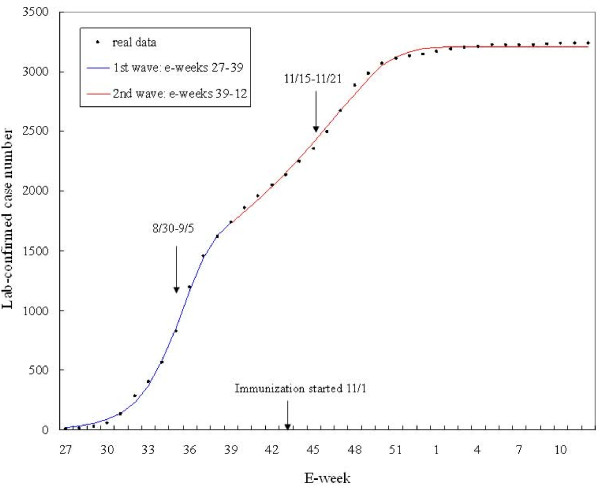
**Model fit for the 2-wave Richards model using weekly confirmed pH1N1 case data by sample receiving week in Taiwan**. The dots are the real cumulative data, the blue curve denotes the first wave, and the red curve denotes the second wave. The arrows indicate the weeks in which turning points had occurred.

We first fit the weekly laboratory confirmed pH1N1 case data by sample receiving week in Taiwan from e-week 27 (6/28-7/4) of 2009 to e-week 12 (3/21-3/27) of 2010. The data fit converges to a two-phase Richard model with the first summer wave spanning e-weeks 29-39 (6/28-9/26) of 2009 and the second fall/winter wave spanning e-week 39 of 2009 to e-week 12 of 2010 (9/20/2009-3/27/2010). The estimation results are given in Table 1 with the model fit shown in Figure 2.

**Table 1:** Model parameter estimates for the Richards model using weekly confirmed pH1N1 case data by sample receiving week in Taiwan from e-week 27 (6/28-7/4) of 2009 to e-week 12 (3/21-3/27) of 2010.

Time Period	**Turning point t**_**i**_ (95% C.I.)	Growth rate r (95% C.I.)	Max case number K^3^ (95% C.I.)	Reproduction number R
e-weeks 27-39	8.50 (7.62, 9.38)	0.50 (0.37, 0.62)	1836 (1676, 1996)	1.14 (1.04, 1.25)
e-weeks 39-12^2^	7.96 (7.42, 8.50)	0.06 (0.05, 0.06)	3214^1^ (3194, 3233)	1.02 (1.01, 1.02)

The first summer wave spanning e-weeks 27-39 of 2009 (6/28-9/26); the second fall/winter wave spanning e-week 39 of 2009 to e-week 12 of 2010 (9/20/2009-3/27/2010); the estimate of effective reproduction number R are obtained by using estimate of T from pH1N1 data of Mexico: T = 1.91 (95% CI: 1.30-2.71) [25].

^1^Estimated max case number for both waves.

^2^e-week 12 of 2010.

^3^The actual confirmed case number (approximated by K in our model) is 1742 during the first wave and 3238 for the two waves.

The turning points for the two waves are estimated at 8.50 weeks after e-week 29 and 7.96 weeks e-week 39, respectively. Subsequently, the weeks in which the turning points for temporal changes in the weekly confirmed pH1N1 case number took place on e-week 36 (8/30-9/5) for the first wave with a 95% CI range of (36.62, 37.38), and on e-week 47 (11/15-11/21) with a 95% CI range of (46.42, 47.50) for the second wave. We note that the above results were obtained by rounding off the estimates to the next largest integer, e.g., e-week 27+8.50 = 35.50 and hence e-week 36 is the week during which the turning point for the first wave occurred, and similarly for the second wave.

To compute the effective reproduction number R, we use the generation time T = 1.91 days (95% CI: 1.30-2.71) for the 2009 pH1N1 in Mexico estimated by Fraser et al. [26]. We note that the given CI's for R_0 _reflect the uncertainty in the generation time T as well as in the uncertainty in the least-squared estimates for r, and does not reflect the error due to the model itself, which is always difficult to measure.

**Figure 3:**
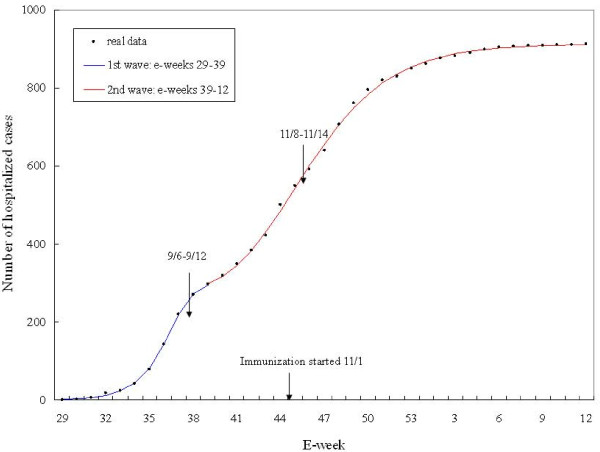
**Model fit for the 2-wave Richards model using weekly pH1N1 hospitalization data in Taiwan; from e-week 27 (6/28-7/4) of 2009 to e-week 12 (3/21-3/27) of 2010**. The dots are the real cumulative data, the blue curve denotes the first wave, and the red curve denotes the second wave. The arrows indicate the weeks in which turning points had occurred.

We also fit the weekly confirmed pH1N1 hospitalization data by hospitalization week in Taiwan from e-week 29 (7/12-7/18) of 2009 to e-week 12 (3/21-3/27) of 2010 to the Richards model. The results are given in Table 2. The data also fit a two-phase Richards model with the first wave spanning e-weeks 27-39 (7/12/09-9/26/09) of 2009 and the second wave from e-week 39 (9/20-9/26) of 2009 to e-week-12 (9/27/09-3/27/10) of 2010 (Figure 3).

**Table 2:** Model parameter estimates for the Richards model using weekly confirmed pH1N1 hospitalization data in Taiwan from e-week 29 (7/12-7/18) of 2009 to e-week 12 (3/21-3/27) of 2010.

Time Period	**Turning point t**_**i**_ (95% C.I.)	Growth rate r (95% C.I.)	Max case number K^3^ (95% C.I.)	Reproduction number R
e-weeks 29-39	7.47 (7.12, 7.82)	1.38 (0.92, 1.83)	304 (290, 319)	1.19 (1.11, 1.27)
e-weeks 39-12^2^	6.63 (5.64, 7.62)	0.38 (0.32, 0.45)	908^1^ (900, 916)	1.03 (1.02, 1.05)

The first wave spans e-weeks 29-39 (7/12/09-9/26/09) of 2009; and the second wave from e-week 39 of 2009 to e-week-12 (9/27/09-3/27/10) of 2010; the estimate of effective reproduction number R are obtained by using estimate of T from pH1N1 data of Mexico: T = 1.91 (95% CI: 1.30-2.71) [25].

^1^Estimated max case number for both waves.

^2^e-week 12 of 2010.

^3^The actual number of confirmed hospitalizations is 297 for the first wave and 910 for the two waves.

The turning points for the weekly confirmed pH1N1 hospitalizations occurred on e-week 37 (9/6-9/12) for the first wave with a 95% CI range of (36.12, 36.82) and on e-week 46 (11/8-11/14) with a 95% CI range of (44.64, 46.62) for the second wave, which were the same weeks as the case number data turning points. The estimate for R using an estimated generation time T for pH1N1 in Mexico [26] is again provided.

**Figure 4:**
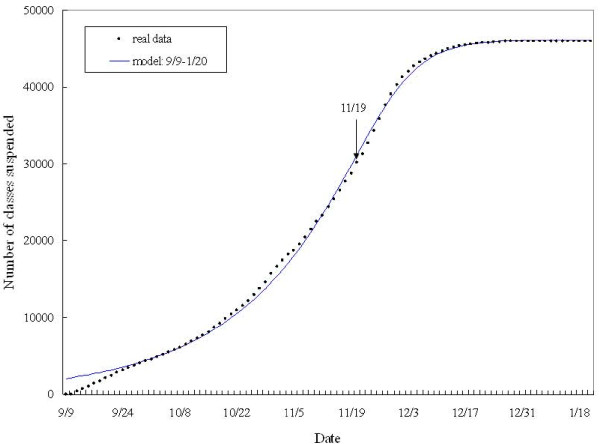
**Model fit for the 2-wave Richards model using daily number of classes suspended due to "325" class suspension policy in Taiwan**. The dots are the real cumulative data and the blue curve denotes the model fit. The arrows indicate the weeks in which turning points had occurred.

**Figure 5:**
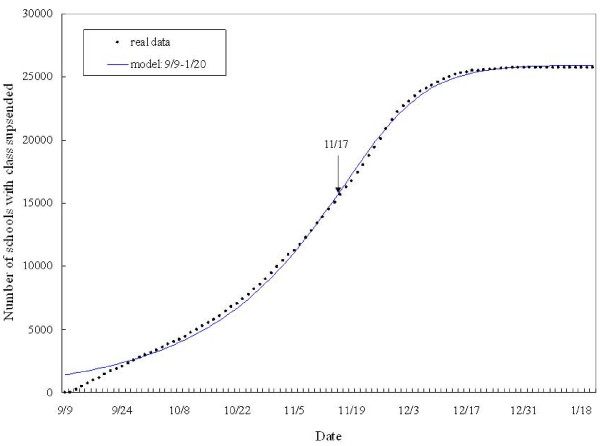
**Model fit for the 2-wave Richards model using number of schools with at least one class suspended in Taiwan; from e-week 29 (7/12-7/18) of 2009 to e-week 12 (3/21-3/27) of 2010**. The dots are the real cumulative data and the blue curve denotes the model fit. The arrows indicate the weeks in which turning points had occurred.

To further analyze and compare our previous results, we also make use of the daily class suspension data in Taiwan from September 9, 2009 to January 20, 2010, which allows us to ascertain the temporal changes in this intervention measure during the time period. Since this dataset started near the end of the first wave, according to our previous results, only one wave was modeled via the Richards model. The estimation results for model fit using the daily class suspension number data as well as the daily number of schools with at least one class suspended are given in Table 3 and Figures 4, 5.

**Table 3:** Model parameter estimates for the Richards model using (I) the daily number of classes suspended and (II) the daily number of schools with at least one class suspended due to the 325 policy in Taiwan during September 9, 2009 to January 20, 2010; the estimate of effective reproduction number R are obtained by using estimate of T from pH1N1 data of Mexico: T = 1.91 (95% CI: 1.30-2.71) [25].

Time Period	**Turning point t**_**i**_ (95% C.I.)	Growth rate r (95% C.I.)	Max case number K (95% C.I.)	Reproduction number R
(I)	50.71^1^ (49.96, 51.46)	0.05 (0.053, 0.057)	46104 (45797, 46410)	1.11 (1.108, 1.112)
(II)	48.81^2^ (47.73, 49.88)	0.05 (0.050, 0.055)	25968 (25755, 26182)	1.11 (1.103, 1.108)

*Max (0, lower bound).

^1^50.71 school days after September 9 corresponds to November 19 as the turning point.

^2^48.81 school days after September 9 corresponds to November 17 as the turning point.

**Figure 6:**
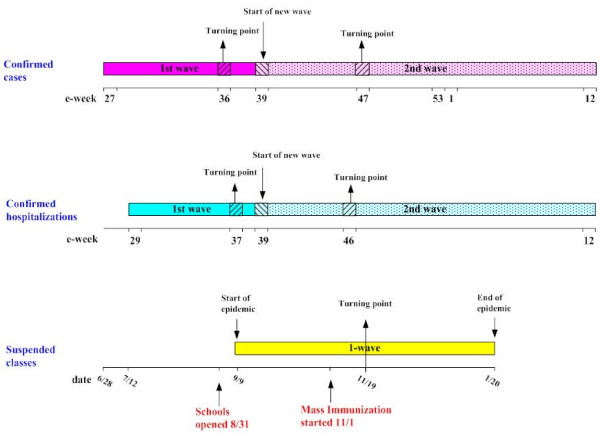
**Chronological timelines of the 2009 pH1N1 epidemic in Taiwan from e-week 27 (6/28-7/14) of 2009 to e-week 12 (3/21-3/27) of 2010, as modeled by the 2-wave Richards model**. The time scales are in e-weeks (top two timelines) or days (bottom timeline). The dotted area denotes the second wave and the shaded areas denote the weeks on which turning points occurred.

**Figure 7:**
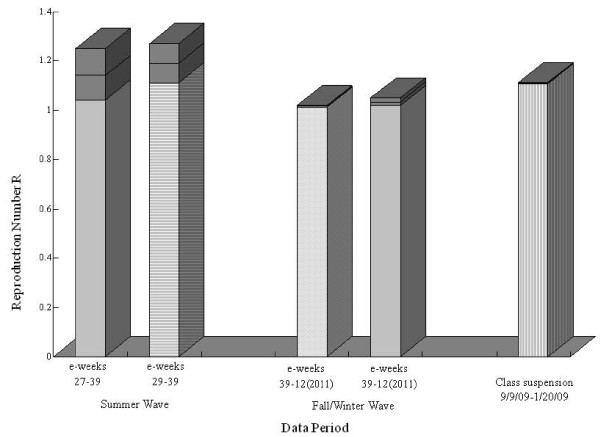
**Effective reproduction numbers R for 2009 pH1N1 during summer of 2009 to early winter of 2010 in Taiwan estimated from the 2-wave Richards model using weekly lab-confirmed case data (unshaded), the weekly confirmed hospitalization data (shaded horizontally), and the daily class suspension data (shaded vertically)**. The upper lightly darkened segments denote the 95% confidence interval of the estimates.

The turning point t_i _is 50.71 (95% CI: 49.96, 51.46) for the daily class suspension number data and 48.81 (95% CI: 47.73, 49.88) for the daily number of schools with at least one class suspended. Since the datasets cover only the school days, we account for the holidays to conclude that 50.71 school days after September 9 corresponds to November 19 (95% CI: November 18-20) as the turning point for the daily class suspension data, while t_i _= 48.81 for the schools with suspension data corresponds to November 17 (95% CI: November 16-18) as the turning point. A graphical illustration of the temporal timelines of the epidemic, as illustrated by the three model fits, is given in Figure 6. Moreover, an illustrative comparison of the estimates for R as obtained by the model fits is also provided in Figure 7. In both Figures 6 and 7, the results from fitting the number of schools with class suspended are omitted for brevity, since they are similar to that of the fitting with class suspension data.

## Discussion

The estimates for effective reproduction number R obtained from the confirmed case and hospitalization data are in good agreement, with R in the range of 1.04-1.27 for the first summer wave during July-September, and 1.01-1.05 for the second wave in fall/winter, using the generation time estimated by [26] for the spring outbreak in Mexico. Serological evidence has indicated that approximately one in every ten persons was infected with the 2009 pH1N1 virus in central Taiwan by April-June [1,27]; hence the estimates using data after July does not yield, and can reasonably be expected to be lower than, the more commonly known *basic *reproduction number R_0_.

A recent modeling study [28] of the 2009 pH1N1 epidemic by geographic region in Mexico reveals a three-wave pandemic, with an initial wave in April-May (Mexico City area), a second wave in June-July (southeastern states), and a geographically widespread third wave in August-December. The estimates for the regional reproduction numbers R were 1.8-2.1, 1.6-1.9, and 1.2-1.3 for the spring, summer, and fall waves, respectively. The second and third waves in Mexico occurred, respectively, one month earlier than the summer (July-early September) and fall/winter (late September-March 2010) waves in Taiwan under study here and exhibit similar decreasing trend, although with higher R.

Transmissibility of the fist pH1N1 wave in Taiwan during the summer in July-September, as measured by R, was lower than that of the earlier spring outbreak in North America [20,26,29,30] and Europe [31], most likely, at least in part, due to decreased social contacts among the population triggered by public awareness of the earlier, well-publicized outbreaks in Mexico and North America as well as the subsequent preemptive government campaign to reduce transmissions. It was also lower than that of the winter outbreak in the Southern Hemisphere around the same time [19,32,33], perhaps attributable to the fact that it was the winter influenza season in the Southern Hemisphere. Moreover, It is lower than the final size estimate of R_0 _(1.87; 95% CI: 1.68-2.06) obtained from serological study of a cohort household population in central Taiwan during the same period of time [1]. However, we note that this disparity is reasonable since the serologic data used for this estimate accounts for the asymptomatic cases among the cohort group. The decreased transmissibility (smaller R) during fall/winter can be reasonably attributed to increased community-wide immunity from the first wave, and perhaps to the 325 class suspension policy initiated in early September before the start of the fall/winter wave.

Significantly higher estimate of R (focused on schoolchildren) in the range of 2.0-2.6 was found for the initial pandemic wave in Japan [34]. Using updated epidemic data and an age-structured model, the same authors also estimated R for the subsequent community-wide wave in Japan in early summer to be much lower (1.21-1.35) [35], although different population and modeling methodology also may have played a role in the decrease in R in subsequent waves. Similar decreases in estimates of reproduction number of 2009 H1N1 when more than one pandemic wave had occurred have been reported in many countries, including Mexico [28], Argentina and Brazil [19], Canada [20], and Japan [34,35]. Furthermore, these studies show that it is not uncommon for multi-wave outbreaks to be more transmissible in a first wave but less widespread with a smaller number of infections (or perhaps limited to a small subpopulation as was in the case of pH1N1 in Japan), when compared to subsequent waves. Moreover, the second wave in Taiwan started shortly after the school opened in September, when additional infections occurring in school settings (as demonstrated by substantial number of class suspensions) contributed to a large number of cases, but perhaps with relatively less per contact transmissibility when compared to household contacts, as it has been reported that sitting next to a case or being the playmate of a case did not significantly increase the risk of H1N1 infection [36].

The estimates for R using laboratory-confirmed case data by sample receiving weeks are slightly lower than those obtained by using confirmed hospitalization data. Although both the confirmed case and hospitalization datasets identify week 39 as the cutoff week for the two waves, the estimates of turning points for each wave differ by about one week when using the two datasets. Since only the more severe confirmed cases were hospitalized, the individuals in the resulting hospitalization time series is a *selected subset *of those in the confirmed case time series. Subsequently, the temporal trends of the two time series might not be closely comparable. However, the cumulative curves in Figures 2, 3, 4, 5 indicate some similarity in the temporal trends of the cumulative data, mainly in the form of the turning points. The reproduction numbers of the two datasets, on the other hand, are indeed comparable since they mostly are generated from the initial growth rates and hence less affected by any selection bias.

The confirmed case data is generated by sampling week, which could be different from the week of symptom onset and hence pose a potential source of some bias in data. However, samples were typically taken when the physicians diagnosed and reported H1N1 cases. We refer to 2003 SARS outbreak in Taiwan, when it was estimated that the onset-to-diagnosis interval is 1.20 days for previously quarantined persons and 2.89 days for non-quarantined persons [37]. Given the similarity in symptoms of SARS and influenza as well as the heightened public awareness due to the world-wide alarm over the seriousness of the pH1N1 pandemic by September, it is more than likely that the time delay from symptom onset to diagnosis (and sample collection) of pH1N1 cases in Taiwan would be no more, if not less, than that of 2003 SARS. Moreover, one would expect that the lesson of SARS and the subsequent efforts by the government to educate has taught the general public in Taiwan to avoid delays in seeking medical care. Subsequently, this delay of one or two days in the *weekly *data can be expected to be most likely not significant. The use of hospitalization data is mainly for the purpose of estimation of reproduction number and comparison with the resulting estimates using the confirmed case data, which is not affected by this delay that might be present in both data.

Estimates of R obtained by using other (larger) estimated generation time in literature result in larger values for R, but generally are well within the ranges of the other studies (see, e.g., [19,20,26,29-32] and Table 2[33]) and hence is omitted for brevity. Note also that the formula for R used here yields an upper bound over all possible distributions for T given the growth rate r, and hence might result in an overestimate of its true value.

In Taiwan, the fall session for kindergarten to high school started on August 31, while the universities started the fall semester two weeks later, around mid-September. Our analysis using the weekly confirmed case and confirmed hospitalization data shows that the initial summer wave of pH1N1 epidemic in Taiwan had peaked by e-week 36-37 (8/30-9/12), around the time schools from kindergarten to grade 12 reopened on August 31. However, a second fall/winter wave of cases started to emerge near the end of September around e-week 39 (9/27-10/3), approximately 4 weeks after the schools reopened, which did not reach its peak until mid-November (e-week 46-47 or 11/8-11/21) and lasted until the turn of the year. It is interesting to note that the state-specific fall pandemic waves in Mexico began 2-5 weeks after school reopened [28], which is consistent with our results on the start of the fall wave in Taiwan. Note that both turning points of the two waves in Taiwan fell on neighboring week using either the lab-confirmed case or hospitalization data. This is reasonable since the hospitalization of confirmed cases and the time that the samples were received by laboratories are closely related, although not necessarily in any particular order.

The class suspension data started on September 9 near the end of the first wave when the earliest class suspension occurred, according to our 2-wave fitting in Tables 1 and 2, hence only one wave was modeled via the Richards model (Table 3). Moreover, November 19 (95% CI: November 18-20) was determined to be the turning point for the daily class suspension data, while November 17 (95% CI: November 16-18) is the turning point for the daily number of schools with class suspended. Both days fall on e-week 47, which coincides with the week where the turning point had occurred as pinpointed by using the confirmed case data and one week after the turning point obtained by using the hospitalization data. It is reasonable to expect the class suspension to take place following the occurrence of case reporting and hospitalization. Moreover, the use of daily data allows a more precise estimation of the turning point.

Also of interest is the possible impact of major intervention measures implemented by the Taiwan government during this time period, which including the aforementioned "325 class suspension" policy and the mass immunization program. The daily number of class suspensions started to increase in early September and continued until late November after the implementation of mass immunization campaign (Figure 1). In particular, the 325 policy, which was designed to minimize the potential social impact of full-scale school closings in the event of a major influenza outbreak in the community; deserve special attention to ascertain its actual effectiveness. In fact, the lower estimates of R for the second wave and for the school closings data might indeed be attributable to the possible effects of school closings after September. However, more detailed class suspension data as well as age-specific epidemic data is needed to further quantify the actual impact or effectiveness of this very unique approach of partial school closure and localized class suspensions on the infections in the school and in the community in a qualitative modeling analysis (see, e.g., [12,13,38]).

## Conclusion

Using routine influenza surveillance data, we modeled the temporal changes of the two waves of pH1N1 epidemic in Taiwan in summer and in fall/winter. The mass H1N1 vaccination program was first initiated sequentially on November 1, where a typical delay of at least two weeks from immunization is needed for protection from the vaccine to take effect in human bodies. Our results suggest that the turning point for the second wave of infections in the fall had occurred around mid-November (e-week 46-47 or 11/8-11/21). Moreover, the class suspension data indicate that the number of class suspensions had peaked by November 20, less than three weeks after the start of mass immunization and most likely before the impact of mass immunizations started to become significant. However, the mass immunization, and perhaps the voluntarily decreased social contacts by the general public in response to the well-publicized mass immunization campaign by the government, could have contributed to the overall mitigation of the disease in the community, as indicated by the early saturation of the winter epidemic by early February. However, this cannot be modeled without detailed vaccination data.

The Richards model considers only the cumulative infected population size with saturation in growth as the outbreak progresses, which can be caused by other factors such as implementation of control measures. Although data by reporting date is often and typically scrambled by artificial factors such as health system alertness, public response, and government responsiveness, the Richards model is able to capture the turning points of outbreaks because they are often results of these artificial factors. We note, however, that the skewness in an epidemic curve, as quantified by the exponent of deviation "a" in the Richards model which describes the curvature of a given cumulative case data, also could conceivably arise from various other intrinsic factors such as spatial heterogeneity and individual heterogeneity in contact (see [39], pp. 281 for example) which is not captured by this simple model.

This type of modeling, although somewhat simplistic and subsequently limited in its quantification of complex factors, nevertheless enables us to ascertain the impact of these artificial factors through the temporal changes of an outbreak, especially in the events when detailed epidemic data describing disease transmissions and other relevant data (such as that of intervention measures in this case) are not readily available for the construction of a complete disease transmission model and the reliable estimation of model parameters, as in this study Moreover, the use of cumulative numbers could often, or at least partially, smooth out stochastic variations that typically occur in epidemic data, and hence the Richards model could be a valuable tool in providing clues to the challenging task of public health policy evaluation and planning.

## Competing interests

The authors declare that they have no competing interests.

## Authors' contributions

YHH conceived and organized the study, carried out the analysis, and wrote the first draft. YHH, KFC, TCL, TNW, CYC, and JWC participated in the study and the interpretation of study findings. KFC participated in the writing of the manuscript. MHL participated in the data collection and analysis. All authors have read and approved the final manuscript.

## Pre-publication history

The pre-publication history for this paper can be accessed here:

http://www.biomedcentral.com/1471-2334/11/332/prepub
